# Designing theoretically-informed implementation interventions: Fine in theory, but evidence of effectiveness in practice is needed

**DOI:** 10.1186/1748-5908-1-5

**Published:** 2006-02-23

**Authors:** Onil Bhattacharyya, Scott Reeves, Susan Garfinkel, Merrick Zwarenstein

**Affiliations:** 1 Department of Health Policy, Management and Evaluation, University of Toronto, Ontario, Canada; 2 Department of Family & Community Medicine, University of Toronto, Ontario, Canada; 3 Centre for Faculty Development, St. Michael's Hospital, Toronto, Ontario, Canada; 4 Wilson Centre for Research in Education, University of Toronto, Ontario, Canada; 5 Sunnybrook and Women's College Health Sciences Centre, Institute for Clinical Evaluative Sciences, Toronto, Ontario, Canada; 6 Knowledge Translation Programme, University of Toronto at St. Michael's Hospital, Toronto, Ontario, Canada

## Abstract

The Improved Clinical Effectiveness through Behavioural Research Group (ICEBeRG) authors assert that a key weakness in implementation research is the unknown applicability of a given intervention outside its original site and problem, and suggest that use of explicit theory offers an effective solution. This assertion is problematic for three primary reasons. First, the presence of an underlying theory does not necessarily ease the task of judging the applicability of a piece of empirical evidence. Second, it is not clear how to translate theory reliably into intervention design, which undoubtedly involves the diluting effect of "common sense." Thirdly, there are many theories, formal and informal, and it is not clear why any one should be given primacy. To determine whether explicitly theory-based interventions are, on average, more effective than those based on implicit theories, pragmatic trials are needed. Until empirical evidence is available showing the superiority of theory-based interventions, the use of theory should not be used as a basis for assessing the value of implementation studies by research funders, ethics committees, editors or policy decision makers.

## Introduction

The Improved Clinical Effectiveness through Behavioural Research Group (ICEBeRG) authors assert [1,2] that a key weakness in implementation research is the unknown applicability of a given intervention outside its original site and problem. They argue that more widely ***applicable ***interventions (and imply that more ***effective ***interventions) should be created by: (1) using explicit behavioral theories to quantitatively characterize the determinants of professionals' behavior choices, (2) identifying predictors that are common across many settings and problems, and (3) designing interventions based on the most powerful predictors. Though this view is logical, it is problematic, and not based on empirical evidence.

### First, the presence of an underlying theory does not necessarily ease the task of judging the applicability of a piece of empirical evidence

Judgment on the wider applicability of a piece of evidence proceeds by induction, and is not mechanistically related to the underlying theory from which grew the empirical study. Behavioral theory is possibly less predictive of behavior than physiological theory is of physiology. It is further diluted in its predictive power by contextual differences, such as health service design and medical cultural differences whose effects on choice cannot be directly translated into the internal psychological forces which are the subject of behavioral theory. We should also bear in mind that the physiological theory predicting a cardio-protective physiological effect for hormone replacement therapy was so convincing that millions of women were prescribed it, but in empirical studies it failed to achieve the predicted benefits, and indeed resulted in substantial harm [3]. Formal theory may be an unreliable predictor of outcome even within the theorized group, and thus a poor framework for extrapolation of outcome to other settings and subjects.

### Secondly, it is not clear how to translate theory rigidly into intervention design

There is no reproducible, algorithmically operationalised process for taking predictor variables from a quantitative theory based descriptive study and turning them into elements of an intervention. Since this process will be diluted by human judgment, which is influenced by many factors other than the theory (i.e., knowledge of context and personal prior beliefs), we believe that theory is contributing less to this part of the process than it appears. Theory could be merely a cover for common sense, or a grounded approach to designing an intervention.

### Thirdly, there are many theories, formal and informal, and it is not clear why any one should be given primacy

Theories overlap and contradict each other. Even theoreticians are forced to distill from the multitude of testable formal theories relevant to professional behavior change a common core of domains; in itself a new, meta-theory, but because of its reverse engineering, based upon little more than common sense [4]. Many formal theories and concepts in the field of psychology had already been described recognizably using lay terms and ideas, suggesting that these ideas are accessible without theories. We live in our own psyche, observe ourselves, reflect on our situation, and ask our colleagues why they make choices. Others observe our choices, directly, through inquiry or by analysis of routine data, and speculate on its determinants. Though not particularly rigorous, all these approaches are plausible sources of informal 'theories.' As such, they can explain professional behavior and inspire ideas for the design of interventions to change behavior, which can then be tested.

### How could we decide whether formal theory offers the best approach for designing interventions to change behavior?

Abstract arguments on this question will continue inconclusively [5]. On the one hand, theory development may lead to a greater meta-understanding and move the field forward. On the other hand, the phenomena being studied may be so complex that all this work will not lead to theories with greater predictive power than implicit theory or "common sense." The exercise may be so time-consuming (e.g., the 20 to 80 years spent conceptualizing cognitive behavioural theory is Eccles et al.'s example [2]) that it may not be a particularly efficient way to proceed. We need an empirical answer to Eccles et al.'s assertion that "better evaluations of what does and does not work in implementation research will only be possible with the explicit use of theoretically informed interventions." We need to know, in practice, whether interventions to change professional behavior, designed using formal theory applied in a predefined and reproducible manner, are more effective at changing the targeted behavior than alternative, less theory bound approaches. Given a sufficient set of replicates, across a reasonable range of settings and professional behavior choices, we can reach an empirical answer. One such randomized trial is underway (TRYME protocol, Francis et al, in submission).

Until there is empirical evidence that interventions designed using theories are generally superior in impact on behavior choice to interventions not so designed, the choice to use or not use formal theory in implementation research should remain a personal judgment. Research funders, ethics committees, systematic reviewers, editors, and policy decision makers should not in any way restrict this choice.

## Competing interests

The author(s) declare that they have no competing interests.

## Authors' contributions

OB wrote the first draft, MZ suggested the idea for the paper and commented on all of the drafts, SR wrote the second draft, and SG modified subsequent drafts. All authors read and approved the final manuscript.
